# Deferoxamine topical cream superior to patch in rescuing radiation‐induced fibrosis of unwounded and wounded skin

**DOI:** 10.1111/jcmm.18306

**Published:** 2024-04-13

**Authors:** Charlotte E. Berry, Darren B. Abbas, Michelle Griffin, Hendrik Lintel, Jason Guo, Lionel Kameni, Andrew A. Churukian, Alexander Z. Fazilat, Kellen Chen, Geoffrey C. Gurtner, Michael T. Longaker, Arash Momeni, Derrick C. Wan

**Affiliations:** ^1^ Hagey Laboratory for Pediatric Regenerative Medicine, Division of Plastic and Reconstructive Surgery, Department of Surgery Stanford University School of Medicine Stanford California USA; ^2^ Department of Surgery The University of Arizona College of Medicine Tucson Arizona USA; ^3^ Institute for Stem Cell Biology and Regenerative Medicine Stanford University Stanford California USA

**Keywords:** cream, deferoxamine, fibrosis, patch, radiation, radiation‐induced fibrosis, wound healing

## Abstract

Topical patch delivery of deferoxamine (DFO) has been studied as a treatment for this fibrotic transformation in irradiated tissue. Efficacy of a novel cream formulation of DFO was studied as a RIF therapeutic in unwounded and excisionally wounded irradiated skin. C57BL/6J mice underwent 30 Gy of radiation to the dorsum followed by 4 weeks of recovery. In a first experiment, mice were separated into six conditions: DFO 50 mg cream (D50), DFO 100 mg cream (D100), soluble DFO injections (DI), DFO 1 mg patch (DP), control cream (Vehicle), and irradiated untreated skin (IR). In a second experiment, excisional wounds were created on the irradiated dorsum of mice and then divided into four treatment groups: DFO 100 mg Cream (W‐D100), DFO 1 mg patch (W‐DP), control cream (W‐Vehicle), and irradiated untreated wounds (W‐IR). Laser Doppler perfusion scans, biomechanical testing, and histological analysis were performed. In irradiated skin, D100 improved perfusion compared to D50 or DP. Both D100 and DP enhanced dermal characteristics, including thickness, collagen density and 8‐isoprostane staining compared to untreated irradiated skin. D100 outperformed DP in CD31 staining, indicating higher vascular density. Extracellular matrix features of D100 and DP resembled normal skin more closely than DI or control. In radiated excisional wounds, D100 facilitated faster wound healing and increased perfusion compared to DP. The 100 mg DFO cream formulation rescued RIF of unwounded irradiated skin and improved excisional wound healing in murine skin relative to patch delivery of DFO.

## 1 INTRODUCTION

Radiation‐induced fibrosis (RIF) constitutes a devastating sequela of radiation therapy, a mainstay in many cancer treatment regimens. While radiation therapy improves cancer survival rates, patients may face chronic tissue stiffness, pain, contracture and non‐healing wounds.^1^ Deferoxamine (DFO) therapy has been at the forefront of novel therapeutics that have been studied to treat RIF.^2^, ^3^, ^4^ DFO, a potent iron‐chelator, has been FDA‐approved for use in the clinical setting and remains the gold standard for hemochromatosis treatment.^5^ While DFO is delivered systemically in clinical settings, recent studies have developed a patch formulation of DFO to administer this drug to a localized area, harnessing DFO's powerful iron chelating effects in a focused region.^6^, ^7^, ^8^, ^9^, ^10^, ^11^, ^12^

Though the cellular mechanism of topical DFO therapy has been studied and its efficacy via patch delivery in treating RIF has been shown in animal studies,^2^, ^6^, ^13^ the logistical obstacles of applying this patch as a daily therapy for a patient with significant cutaneous damage following radiation may present a translational challenge. Irradiated skin often undergoes a substantial amount of desquamation following radiation exposure, making the use of an adhesive to maintain direct contact between the DFO patch and the affected area difficult and even painful for patients.^14^ Additionally, radiation is often applied in topographically heterogeneous anatomical regions, such as the head and neck, breast and underarms which creates an additional challenge for maintaining consistent application of an adhesive dressing.^15^ Finally, when surgical incisions and wounds occur on irradiated skin, specific application to a wounded area is complicated by the predetermined dimensions of the patch formulation.

In burn patients, the development of cream and ointments has helped significantly to improve the comfort and care that patients receive.^16^, ^17^, ^18^ These have been used to specifically treat radiation burns^19^ as they create an occlusive protective layer over the skin, thus preventing heat and water loss.^20^, ^21^ With this understanding, a cream formulation of DFO was developed to avoid additional dressings and facilitate improved ease of use and comfort for patients affected by RIF. In this study, we aimed to investigate the efficacy of DFO cream compared to previously studied DFO injection and patch delivery treatment modalities in the setting of unwounded and wounded irradiated skin.

## 2 METHODS AND MATERIALS

### 2.1 DFO cream production

Since DFO is hydrophilic, the development of the cream formulation involved incorporating Transcutol P (Gattefosse) to create emulsions which allow for effective penetration of the stratum corneum layer of the skin. Transcutol P is a highly purified form of diethylene glycol monoethyl ether (DEGEE) that serves as a safe solubilizer and enhancer for transdermal drug delivery.^22^ Transcutol P increases drug solubility in the vehicle (water) and penetration through the stratum corneum without disrupting physiologic lipid bilayer skin structures. Transcutol P has been approved by FDA as an inactive ingredient and has already been used in a variety of FDA approved human and veterinary drug products.^22^ Hydroxyethylcellulose was used as a gelling agent and mixed with water before finally being mixed with Transcutol P and DFO overnight in an overhead mixer to create the emulsion. The DFO cream was found to release approximately 46% at 6 h and approximately 83% after 24 h.

### 2.2 Irradiated animals without excisional wounding

**FIGURE 1:**
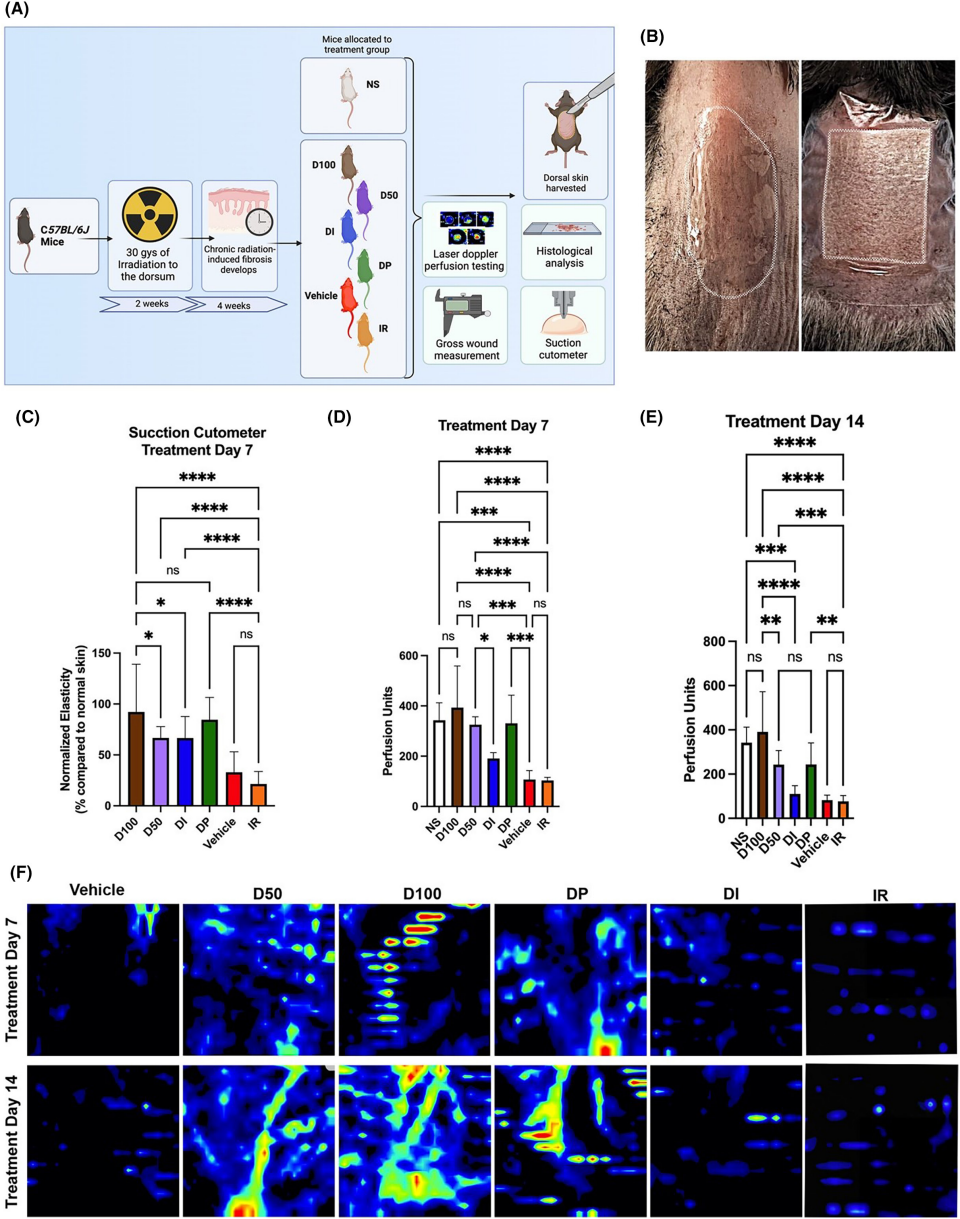
(A) Schematic overview of experimental design demonstrating timeline from 2 weeks of radiation wounding, 4 weeks of recovery, and 2 weeks of treatment before tissue was harvested. (B) Representative image of deferoxamine (DFO) cream therapy (left) and DFO patch therapy held in place with a Tegaderm dressing (right) on irradiated dorsal murine skin. (C) Normalized elasticity measured with suction cutometer demonstrated that control cream had least elastic skin compared to non‐treated, irradiated skin, while all DFO treatment conditions improved skin elasticity. D100 (****p < 0.0001) and DP (****p < 0.0001) treatment modalities had the most improvement in skin elasticity, though D50 (**p < 0.01) and DI groups (**p < 0.01) displayed some improvement in elasticity in irradiated unwounded skin. (D) Statistical analysis of laser Doppler measurements at day 7 of treatment show that D50 (**p < 0.01), D100 (***p < 0.001), and DP (**p < 0.01) all had significantly improved perfusion units compared to IR control or DI group. (E) By day 14 of treatment, IR control and DI groups had lost a significant amount of cutaneous perfusion by laser Doppler. D50 (*p < 0.05) and DP (*p < 0.05) showed significant improvement compared to IR control or DI conditions, but D100 (****p < 0.0001) retained the most cutaneous perfusion after 14 days of therapy, most closely resembling normal skin perfusion. (F) Laser Doppler images of all conditional groups at day 7 and day 14 of treatment. At day 7, DFO 50 cream (D50), DFO 100 cream (D100), and DFO patch (DP) appeared to have some improvement in cutaneous perfusion compared to IR control. DFO injection (DI) showed similar perfusion measurements as IR control. At day 14, D100 displayed a significant increase in cutaneous perfusion compared to even D50 or DP. However, these three groups still demonstrated significantly higher laser Doppler measurements than IR control. IR control and DI maintained similar laser Doppler measurements even after 14 days of therapy.

Six‐week‐old adult C*57BL/6J* female mice (Strain #: 000664, Jackson Laboratory, Bar Harbour, ME) were used (*n* = 30), with five mice allocated to each experimental group. For the chronic RIF experiment, mice were divided into six groups: (1) irradiated and treated with DFO 50 mg cream (D50), (2) irradiated and treated with DFO 100 mg cream (D100), (3) irradiated and treated with soluble DFO injection (DI), (4) irradiated and treated with DFO 1 mg patch (DP), (5) irradiated and treated with DFO‐free cream (Vehicle), and (6) irradiated with no treatment (Figure 1A). Using a previously described protocol for dosage and schedule, mice were irradiated with a total of 30 Greys (Gy) external beam radiation to the dorsum, delivered as six fractionated doses of 5 Gy every other day over the course of 12 days total (Kimtron Polaris SC‐500; Kimtron Inc., Oxford, CT), followed by 4 weeks of recovery to allow for the development of fibrosis.^4^, ^23^, ^24^ Lead shielding was used to isolate exposure to only the dorsa. All experiments were performed under an approved APLAC protocol (APLAC #31212) in accordance with the Stanford University Animal Care and Use Committee Guidelines. Five additional mice were used as non‐irradiated, normal skin (NS) controls. Mice were maintained at the Stanford University Research Animal Facility in sterile micro‐insulators and were given water and rodent chow ad libitum, in accordance with Stanford University guidelines.

### 2.3 DFO treatment protocol

After 4 weeks of recovery following radiation, mice in treatment groups received 2 weeks of daily treatment. The cream formulations (TauTona Group, Redwood City, CA) were created at a concentration of 50 mg DFO per 15 g of cream (D50) and 100 mg DFO per 15 g of cream (D100). The cream was delivered in 450 mg aliquots and spread in a 1.5 × 2 cm rectangular area to cover the entire irradiated field of dorsal skin. As previously described, the DFO patch formulation (TauTona Group, Redwood City, CA) delivered 1 mg DFO per 1 cm^2^.^2^, ^6^, ^13^ A 1.5 × 2cm rectangular section of the patch was created and placed directly onto the irradiated dorsal skin and fixed into place with a Tegaderm (3 M) dressing (Figure 1B). The DFO injection (Selleckchem) formulation was created from DFO mesylate dissolved in sterile phosphate‐buffered saline at a concentration of 10 mg/mL. 300 μL of this DFO solution was injected subcutaneously over the entirety of the irradiated dorsal skin with a 28‐gauge needle and 0.3 mL syringe as previously described.^13^

### 2.4 Irradiated animals with excisional wounding

To study the efficacy of DFO treatment in chronically irradiated excisional wounds, six‐week‐old adult C*57BL/6J* female mice (*n* = 20) were divided into four experimental groups (*n* = 5/group): (1) irradiated and treated with DFO 100 mg cream (W‐D100), (2) irradiated and treated with DFO 1 mg patch (W‐DP), (3) irradiated and treated with DFO‐free cream (W‐Vehicle) and (4) irradiated with no treatment (W‐IR). Mice underwent radiation treatment with 30 Gy, as described above, and were then allowed to recover for 4 weeks before the creation of excisional wounds.

Based on a well‐established silicone‐stented excisional wound model, four excisional wounds were created on the dorsal skin of each mouse.^13^ The mice were anaesthetised using 2% isoflurane at a flow rate of 2 L/min. Dorsal skin hair was removed using an electric razor and chemical depilatory cream, the skin was cleaned using an alcohol swab and two wounds were placed on either lateral side of the dorsal skin. The spine was used as a dividing line and the 6 mm diameter wounds were evenly spaced, with at least 2 cm of space between their outer edges. After the wounds were made, they were stented open with silicone rings which were fastened with glue (Gorilla Glue Co., Cincinnati, OH) and sutured with 5–0 nylon (Covidien, Dublin, Ireland) to ensure the wounds did not close by contraction. For pain management, Buprenorphine SR (1 mg/kg) was administered once directly following anaesthetic induction and then every 2–3 days as needed following the procedure.

Immediately after wounding, group‐specific treatment was applied and the dorsum was wrapped with Tegaderm dressing (3 M, Saint Paul, MN). For wounded groups, the 1.5 × 2cm DFO patch was applied directly to the irradiated skin with the wound exposed in the center of the silicone ring. Cream was applied in an even layer over the same area in 450 mg aliquots. Every other day, the dressings were changed, wounds were measured, photos were captured, and the treatment was reapplied until wound closure.

Additionally, wounds in non‐irradiated NS of five mice were created as controls with no DFO treatment (W‐NS). Experiments were performed under an approved APLAC protocol (APLAC #31212) in accordance with the Stanford University Animal Care and Use Committee Guidelines. Mice were maintained at the Stanford University Research Animal Facility in sterile micro‐insulators and were given water and rodent chow ad libitum, in accordance with Stanford University guidelines.

### 2.5 In vivo tissue vascularity

A PeriScan PIM 3 (Perimed, Järfälla, Sweden) laser Doppler was used to scan for perfusion within the dorsal skin in vivo. Scans were performed at a minimum of weekly intervals during the treatment period. The mice were acclimatized to the temperature and humidity of the laser Doppler chamber for 1 h prior to scanning. The mice were placed under inhaled anaesthesia with 1%–3% isoflurane for the duration of the procedure. Any surface debris was removed from the dorsum with sterile alcohol prep pads (Fisherbrand, Ottawa, Ontario). The laser Doppler chamber was devoid of all ambient light, and the entire irradiated area of the mice was scanned in duplicates.

### 2.6 Tissue harvest

After 2 weeks of treatment, the irradiated, non‐excisionally wounded mice were sacrificed, and dorsal skin was harvested. Mice with excisional wounds were harvested after wound closure. Irradiated wounds that were not closed after 23 days following excisional wounding were designated as ‘non‐healing’ based on prior studies which have indicated that re‐epithelialization no longer progressed past this time point.^13^, ^25^ Specimens for histology were fixed in 10% neutral buffered formalin (ThermoFisher Scientific, Waltham, MA) at 4°C for 18 h, washed with phosphate‐buffered saline, dehydrated in gradients of alcohols, and embedded in paraffin blocks for sectioning.

### 2.7 Dermal thickness and collagen density analysis

For assessment of dermal thickness, 8 μm sections of dorsal skin specimens were stained with Haematoxylin and Eosin (H&E) (Cat#H‐3502; Vector Laboratories, Burlingame, California), and for assessment of collagen density, specimens were stained with Masson's Trichrome (TC) (ab150686; Abcam, Waltham, MA). These specimens were imaged at 10X magnification using a Leica DMI4000 B (Leica Microsystems, Wetzlar, Germany). Integrated density measurements of stained collagen were measured using the ImageJ colour deconvolution plugin. Blue pixel count was quantified through ImageJ using a Colour Detect macro.

### 2.8 Ex‐vivo tissue vascularity

Immunohistochemistry was performed on 8 μm sectioned paraffin slides which were blocked with 1X Powerblock (HK083‐50 K; Biogenex, Fremont, CA) and incubated for 1 h at 37°C with the relevant primary antibody for 1 h at 37°C. Specimens were washed in phosphate‐buffered saline (10,010,023; Gibco®, Dublin, Ireland) and incubated with a secondary antibody for 1 h at 37°C. Specimens were then washed in phosphate‐buffered saline (10,010,023; Gibco®, Dublin, Ireland) and mounted onto glass slides in DAPI Fluromount‐G (0100–01; SouthernBiotech, Birmingham, AL). Fluorescent images of the dermal layer were taken using an SP8 inverted confocal microscope (Leica Microsystems, Wetzlar, Germany) at 20X magnification (25 images per condition). The percentage of stain‐positive pixels was calculated on each image using ImageJ with a Colour Detect macro to quantify the relevant number of pixels.

### 2.9 CD31

Unconjugated anti‐CD31 (PECAM) (Ab28364; Abcam, Waltham, MA) at a 1:100 dilution in 0.1X Powerblock was used as the primary antibody. Alexa Fluor 594 conjugated secondary antibody (Ab150080; Abcam, Waltham, MA) was used as the secondary antibody.

### 2.10 8‐Isoprostane

Unconjugated anti‐isoprostane (8‐iso‐PGF2a) (MBS621657; MyBioSource, San Diego, CA) at a 1:100 dilution in 0.1X Powerblock was used as the primary antibody. Alexa Fluor 430 conjugated secondary antibody (Ab150080; Abcam, Waltham, MA) was used at the secondary antibody.

### 2.11 Machine‐learning algorithm analysis of extracellular matrix architecture

Paraffin sections of skin specimens were stained with Picrosirius Red (ab246832; Abcam, Waltham, MA) per the manufacturer's protocol. A minimum of 100 images per condition were captured at 40X magnification with the Leica DMI4000 B (Leica Microsystems, Wetzlar, Germany) inverted microscope using a polarizing filter. Polarized images of Picrosirius Red‐stained slides were colour deconvoluted, binarized, and skeletonized using a MATLAB algorithm as previously described.^4^, ^13^, ^23^, ^26^ 294 parameters of red and green polarized collagen fibres were extracted (including brightness, number, length, width, persistence, angle, branch points, Euler number, extent, perimeter, solidity, eccentricity, equivalent diameter, etc.), measured, and reduced by Uniform Manifold Approximation and Projection (UMAP) to generate two‐dimensional plots to visualize collective differences in the collagen fibre network patterns between groups. This analysis facilitated a more comprehensive quantified comparison of extracellular matrix features between experimental conditions.

### 2.12 Biomechanical skin testing

Biomechanical skin testing was performed on day 14 of treatment for all unwounded irradiated groups following laser Doppler scans, but before tissue harvest. The skin was cleaned of all debris using sterile alcohol prep pads. The mice were kept anaesthetised with inhaled isoflurane 1%–3%. A Dual MPA 580 suction cutometer device (Courage + Khazaka, Köln, Germany) with a 2 mm probe was placed directly onto the dorsal skin and skin elasticity was measured as previously described.^27^

In all wounded groups, harvested wounds underwent mechanical testing using a servohydraulic MTS 858 Bionix device (MTS Systems Inc., Minneapolis, MN) configured with linear and rotary variable differential transducers and a 3.3 Kip tensile load cell. The wound specimens were secured in clamps on opposite ends of the healed wound and axial load was applied at a defined strain rate of 100 mm/min. Testing progressed until the wound tissue separated, and Young's modulus and stiffness were determined.

### 2.13 Statistical analysis

Parametric analyses were performed using two‐tailed Student's *t*‐tests for comparisons between two groups, and one‐way analysis of variance (ANOVA) followed by post‐hoc analysis using Tukey's multiple comparisons test for multiple groups. Nonparametric analyses were performed with the Kruskal‐Wallis test with post‐hoc Dunn's testing to compare means across groups. GraphPad Prism (GraphPad Software, Inc., San Diego, CA) was used to perform all statistical analyses. A value of *p* < 0.05 was considered significant.

## 3 RESULTS

### 3.1 DFO improves elasticity in chronically irradiated skin

After mice underwent irradiation and developed chronic fibrosis over a four‐week recovery period, treatment with DFO was initiated (Figure 1A,B) In irradiated skin, DFO administration improved elasticity **(**Figure 1C). Measured via suction cutometer, skin stiffness in D50 and DI groups was found to improve, with D100 and DP demonstrating the most improvement in elasticity. However, amongst the irradiated DFO‐treated groups, there were no significant differences in skin elasticity aside for D100, which exhibited significantly improved skin biomechanics compared to D50 and DI (Figure 1C).

### 3.2 DFO cream improves perfusion in chronically irradiated skin and wounds

To determine the amount of vascularity in the irradiated fields in vivo, laser Doppler scans were performed to analyse dermal perfusion. In irradiated skin, laser Doppler scans at day seven of treatment demonstrated a significant improvement in dermal perfusion when DP, D50 and D100 were compared to the IR. A small increase in perfusion was also noted in the DI group, but this was not significantly different from IR. When compared to NS at this time point, DP, D50 and D100 did not show any difference in laser Doppler perfusion, while DI had significantly lower perfusion (Figure 1D). After 14 days of treatment, a small increase in perfusion was again noted with DI, but was not significantly different compared to IR. However, the groups DP, D50 and D100 all demonstrated improved perfusion compared to IR and continued to not significantly differ from NS for any of those three conditions. No significant difference in perfusion was found between Vehicle and IR at either time point (Figure 1D–F).

### 3.3 Radiation‐induced changes in dermal architecture following radiation are improved with DFO cream treatment

**FIGURE 2:**
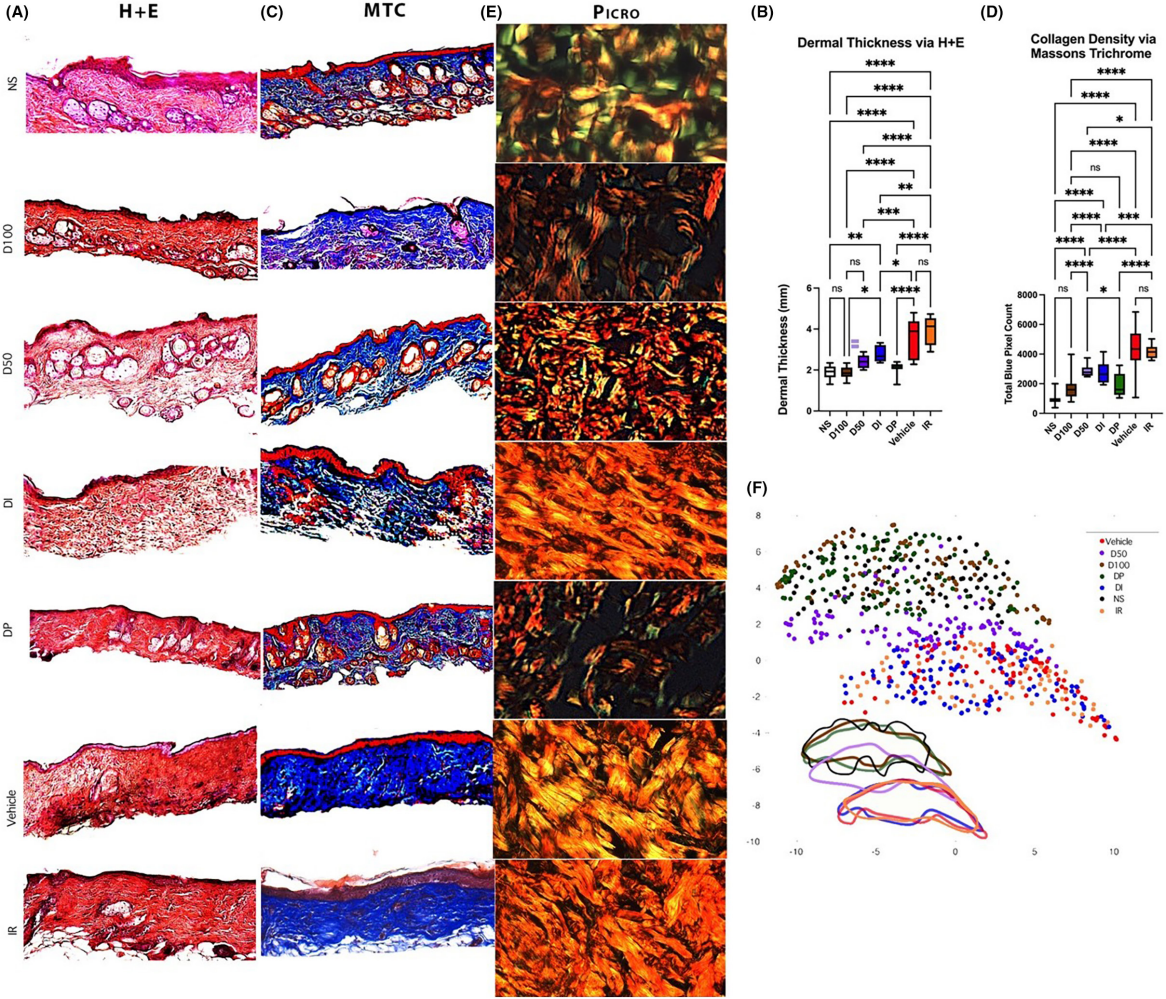
(A) Histological representation of Haematoxylin and Eosin (H&E) staining for all treatment conditions. Grossly, IR, Vehicle and DI demonstrated significant loss of hair follicles and sebaceous glands following radiation wounding compared to normal skin. (B) Quantification of dermal thickness on H&E stains. DI treatment was able to decrease dermal thickness (*p < 0.05) compared to IR, while D50 decreased dermal thickness more (**p < 0.001). D100 and showed the most dramatic improvement in dermal thickness compared to IR (****p < 0.0001 respectively) while simultaneously being the only treatment modalities that did not show any significant difference in dermal thickness compared to normal skin (*p* > 0.99 and *p* = 0.91 respectively). Dermal thickness between D100 and DP was also not significantly different (*p* = 0.95), while thickness between D50 and DI was also not significantly different (*p* = 0.63). However, dermis of DI‐treated mice was significantly thicker than D100 or DP mice (**p < 0.01). (C). Histological representation of Masson's Trichrome (TC) staining for all treatment conditions. (D) Improvements in collagen density within dermis were much more pronounced for all DFO treatment conditions compared to Vehicle and IR (****p < 0.0001). Amongst DFO treatment conditions, DP and D100 demonstrated greatest improvement in collagen density and closely resembled normal skin (*p* = 0.08 and *p* = 0.11, respectively). DP and D100 were similar to each other when analysing collagen density levels (*p* = 0.91). (E) Histological representation of Picrosirius Red staining for all treatment conditions. (F) Proprietary machine‐learning algorithm analysis of Picrosirius Red images plotted these data within 2‐dimensional space utilizing a Uniform Manifold Approximation and Projection (UMAP) for dimension reduction. UMAP visualization of dermal extracellular matrix shows a significant clustering of control and DI architecture into a similar space, clustering of D100/DP/NS into a similar space, and D50 clustering in an intermediary space between other clusters.

The dermal thickness in irradiated skin significantly decreased with all forms of DFO administration, as determined on H&E staining. This decrease was modest in the DI group, with further improvement in dermal thickening with the D50 group and the most significant improvements in dermal thickness exhibited by the D100 and the DP cohorts relative to IR animals. However, when compared to NS, the only two treatment conditions with a similar dermal thickness were D100 and DP (Figure 2A,B). Not only was there an improvement in dermal thickness with DFO treatment, but the density of collagen on Masson's TC within the dermis of irradiated skin was also decreased with DFO. In relation to the IR control, all formulations of DFO including D50, D100, DP and DI decreased the collagen density within the dermis. DP and D100 again demonstrated greater ability to decrease collagen density compared to D50 or DI. The collagen density between D100 and DP was not significantly different, nor was either condition significantly different from the collagen density seen in NS (Figure 2C,D). The similarities in dermal architecture between normal unwounded skin and the irradiated skin treated with D100 and DP persisted when polarized Picrosirius Red images were analysed by a machine learning‐based algorithm for ultrastructural features. This algorithm identified a significant overlap between the IR control and DI groups, with D50 collagen ultrastructure exhibiting a transitional state between clusters (Figure 2E,F). Across all histological parameters, no significant differences were measured between the two control groups (Vehicle and IR).

**FIGURE 3:**
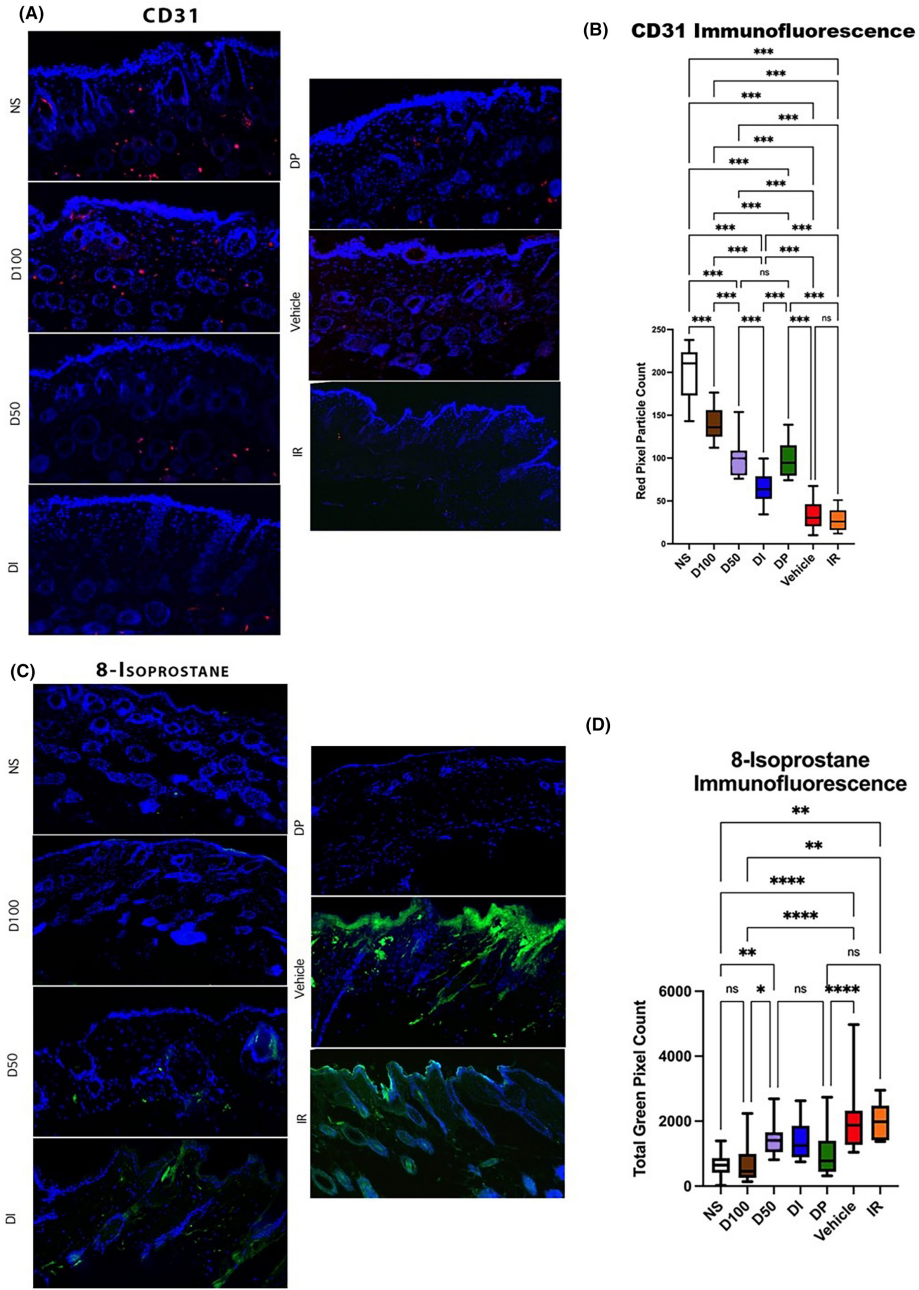
(A) Representative images of CD31 immunofluorescent staining within all treatment conditions. CD31 was visualized as red staining on these images while DAPI was seen as blue. (B) Largest amount of vascularity was seen within unwounded dermis of normal skin, while IR and Vehicle had least amount of vascularity present by end of experimental timeline. Vascularity was best preserved with D100 treatment (***p < 0.001) which was closest to normal skin. Vascularity was least preserved with DI treatment, though amount of CD31 staining present was still significantly higher than IR (***p < 0.001). Both D50 and DP moderately improved vascularity at a similar efficacy (*p* = 0.99), but both still had significantly less CD31 staining present at end of experimental timeline compared to D100 (both ***p < 0.001). (C) 8‐isoprostane immunofluorescent staining, a marker of oxidative damage, was displayed as green stain, while DAPI was represented with blue staining on these representative images. (D) IR and Vehicle cohorts had most oxidative damage with highest amount of 8‐isoprostane staining, and, though 8‐isoprostane staining was slightly reduced with DI treatment, it was not by a significant amount (*p* = 0.18). D50 therapy was able to significantly decrease amount of oxidative damage compared to IR mice (*p* < 0.05), while D100 and patch therapies were most effective at mitigating oxidative damage (both ****p < 0.0001). D100 and DP treatments were both able to prevent oxidative damage to extent that 8‐isoprostane levels were equivalent to those seen in normal skin (*p* = 0.99 and *p* = 0.36, respectively).

To histologically evaluate vascularity within irradiated skin, immunofluorescent staining for CD31 was performed. Decreased staining was seen in the IR group, and much greater CD31 staining was seen in the D50, D100, DP and DI groups, corresponding with our prior in vivo perfusion findings with the laser Doppler. Of note, D100 had significantly greater CD31 staining than the D50 or DP conditions, while the D50 and DP treated animals were not significantly different in this regard. Though significant improvement was seen in vascularity compared to the IR control group, D100 CD31 staining was significantly less than that seen in NS (Figure 3A,B).

To evaluate DFO effects on oxidative stress, staining for 8‐isoprostane, a prostaglandin‐like compound formed from free radical‐catalysed peroxidation of membrane fatty acids, was performed. No difference in staining was noted between Vehicle and IR control, while D50 had some reduction in 8‐isoprostane staining. Reduced staining was also appreciated for DI but was still significantly greater than NS. Both D100 and DP were effective in reducing oxidative stress, as measured by 8‐isoprostane staining, compared to Vehicle and IR groups, with levels similar to NS. No significant difference was noted between D100 and DP (Figure 3C,D).

### 3.4 DFO cream hastens wound healing and decreases incidence of non‐healing

**FIGURE 4:**
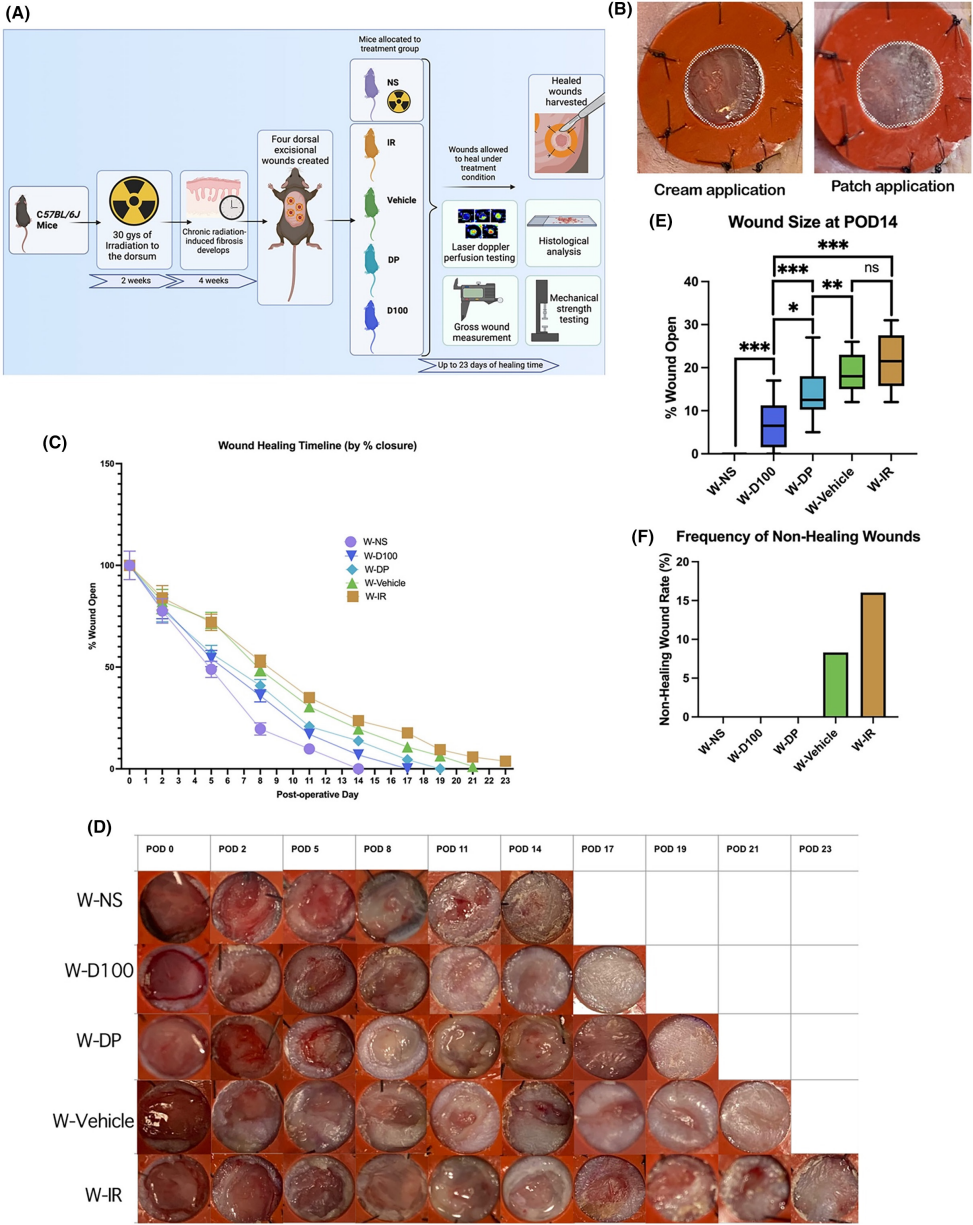
(A) Schematic overview of experimental design demonstrating timeline from 2 weeks of radiation, 4 weeks of recovery, and creation of excisional wounds on dorsum. Laser Doppler perfusion testing and gross wound measurements were taken during wound healing process, and histologic and mechanical stress testing were performed on harvested tissue. (B) Representative image of deferoxamine (DFO) cream therapy (left) and DFO patch therapy (right) on excisional wound model using irradiated dorsal murine skin. (C) Wound size quantification showed accelerated wound closure for W‐D100 and W‐DP compared to W‐Vehicle and W‐IR wounds, but slower compared to W‐NS. (D) Representative images of healing progression of excisional wounds by treatment condition. (E) Wound size at POD 14 showed statistically significant smaller wounds in W‐D100 (***p < 0.001) and W‐DP (**p < 0.01) groups compared to W‐IR wounds, while nonirradiated wounds had closed at this timepoint. (F) W‐Vehicle and W‐IR untreated wounds had a higher rate of non‐healing than DFO‐treated irradiated wounds at POD 23. Wounds in groups W‐NS, W‐D100 and W‐DP all closed.

To investigate the effects of DFO cream on wound healing, four excisional wounds were created in the dorsa of mice following radiation treatment and four‐week recovery for development of chronic fibrosis (Figure 4A). Based on results from DFO treatment of irradiated mice, the most efficacious treatments (D100 and DP) were further explored in our irradiated excisional wounding experiments (Figure 4B). Wounds treated with DFO in groups W‐D100 and W‐DP healed at days 17 and 19, respectively (Figure 4C,D). These time points were comparatively faster than irradiated wound control groups, which healed on day 21 for W‐Vehicle and day 23 for W‐IR, while NS (W‐NS) healed the fastest on day 14. At day 14, wound size in DFO‐treated groups W‐D100 and W‐DP were significantly smaller than irradiated control groups W‐Vehicle and W‐IR. Interestingly, W‐D100 wounds were also significantly smaller than W‐DP wounds on day 14. No significant difference was found in wound size at day 14 between W‐Vehicle and W‐IR (Figure 4E). Incidence of non‐healing wounds was found to be 16% in the W‐IR group and 8.3% in the W‐Vehicle group. No other groups exhibited non‐healing wounds (Figure 4F).

### 3.5 DFO treatment of chronically irradiated excisional wounds results in features of NS scarring histologically

**FIGURE 5:**
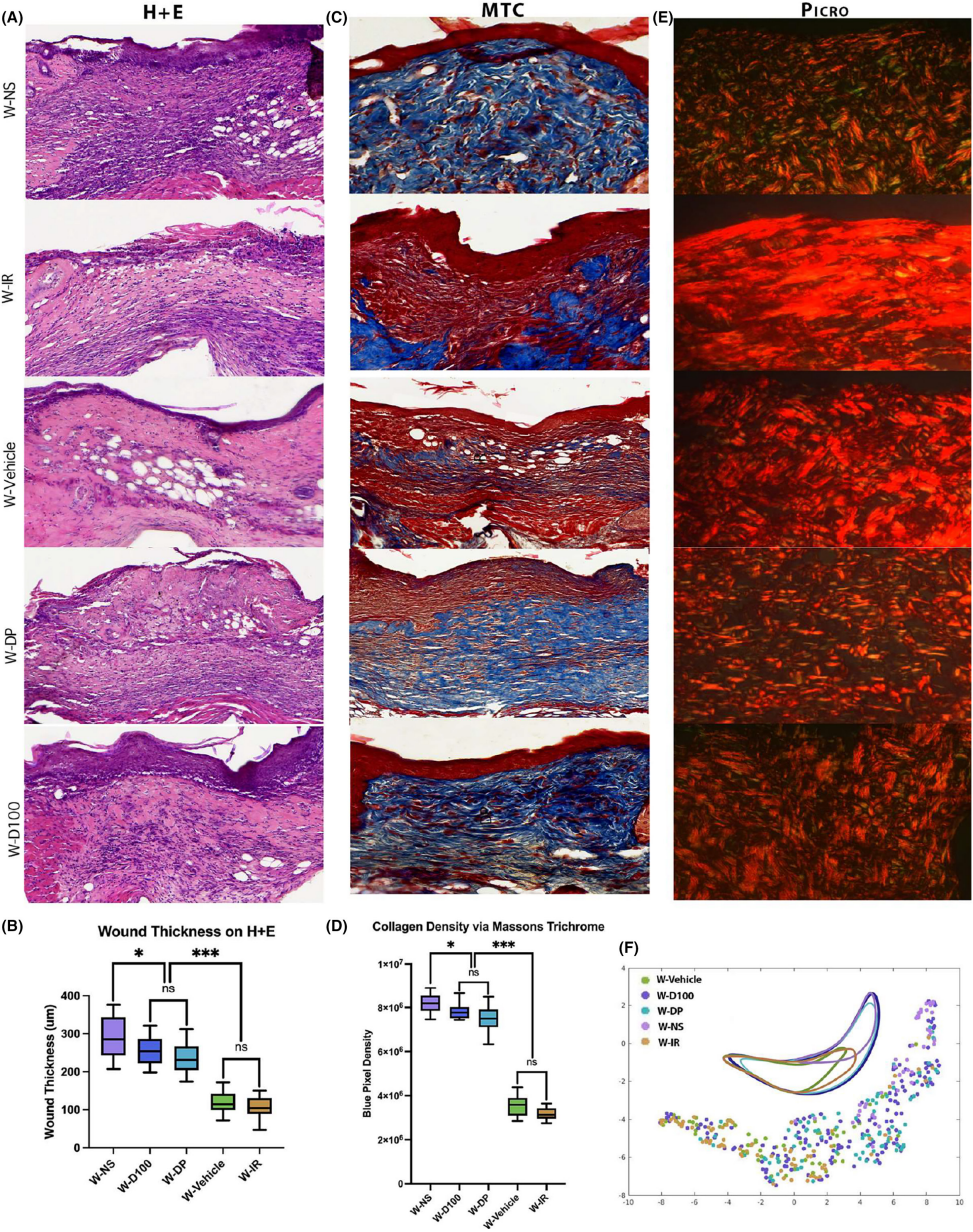
(A) Histological representation of Haematoxylin and Eosin (H&E) staining for all treatment conditions. (B) Quantification of dermal thickness for each wounded treatment group via H&E staining demonstrated no significant difference between W‐D100 and W‐DP treatment groups or W‐Vehicle and W‐IR control groups. Treatment groups demonstrated less dermal thickening within wound than W‐NS (*p < 0.05) and more thickening than control groups (***p < 0.001). (C) Histological representation of Masson's Trichrome (MT) staining for all treatment conditions. (D) Quantification of blue pixel density demonstrated no significant difference between W‐D100 and W‐DP treatment groups or W‐Vehicle and W‐IR control groups. Both treatment groups demonstrated less blue pixel density within wound than W‐NS (*p < 0.05) and more density than control groups (***p < 0.001). (E) Histological representation of Picrosirius Red staining for all treatment conditions. (F) UMAP visualization of dermal extracellular matrix showed a significant clustering of W‐Vehicle and W‐IR architecture into a similar space. W‐D100 and W‐DP appear to share space with both aforementioned control groups and W‐NS.

In excisionally wounded skin, the same histological stains and outcome measures were used to analyse changes between treated and untreated wounds. Using H&E staining, healed wound thickness was measured to be the largest in W‐NS. Both groups treated with DFO, W‐D100 and W‐DP showed thinner healed wounds than W‐NS, although these wounds were substantially thicker than those from the W‐Vehicle and W‐IR groups. No significant difference in thickness was found histologically between W‐D100 and W‐DP; nor were there any differences between the W‐Vehicle and W‐IR wounds (Figure 5A,B). Collagen density determined by TC stain demonstrated the same relationships between groups, with a higher collagen density observed in W‐NS, W‐D100 and W‐DP groups and a lower collagen density observed with irradiated control groups W‐Vehicle and W‐IR (Figure 5C,D). As in irradiated unwounded skin, computational analysis determined that there was significant overlap between the irradiated control groups W‐Vehicle and W‐IR, indicating similar matrix ultrastructural features (Figure 5E,F). In contrast, wounds in the W‐D100 and W‐DP groups were found to share more features with the W‐NS group.

### 3.6 DFO improves biomechanical stiffness of scars formed from excisional wounds

**FIGURE 6:**
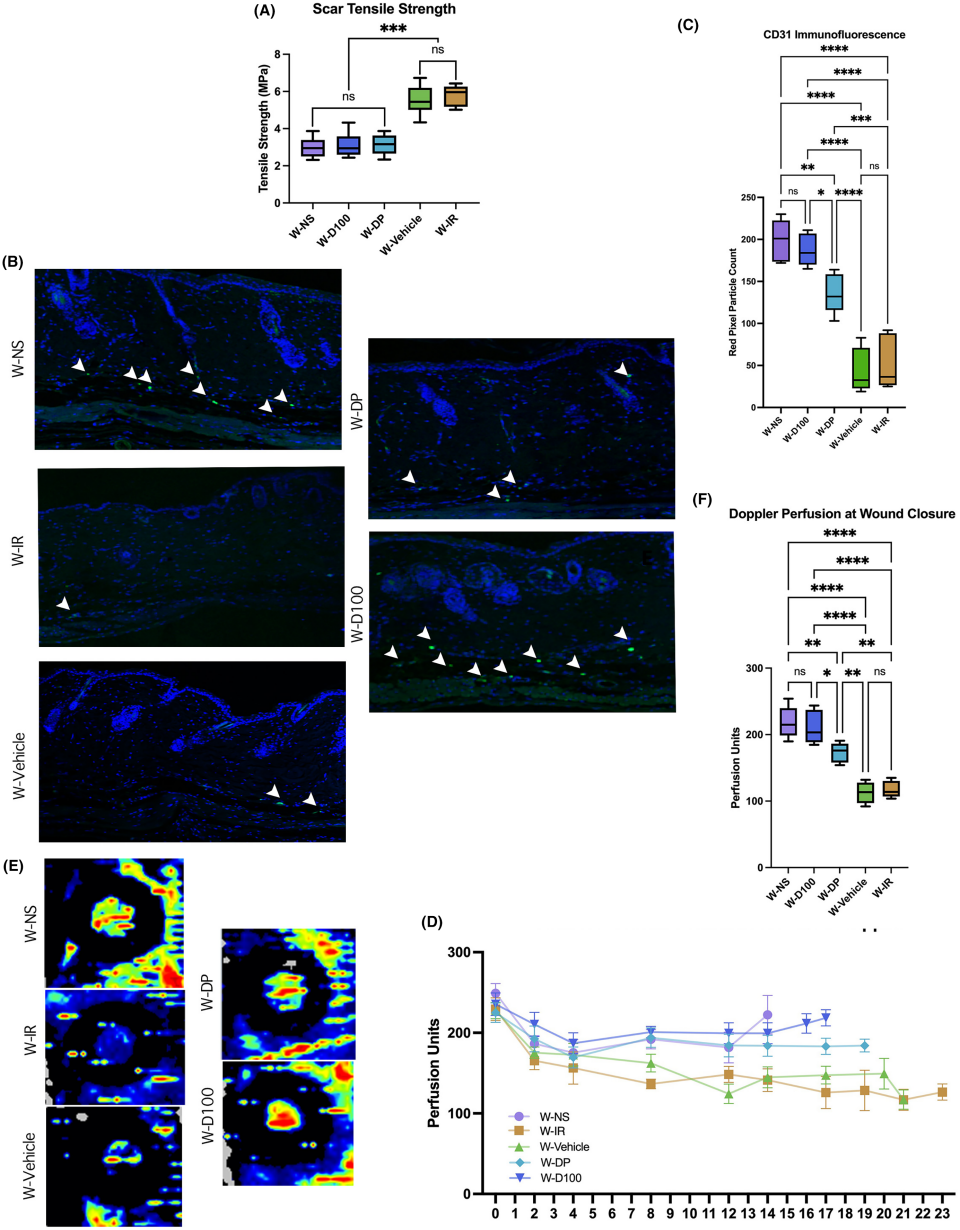
(A) Stiffness testing in MegaPascal (MPa) units demonstrated no significant difference between W‐NS, W‐D100, and W‐DP. No significant difference was measured between control groups W‐Vehicle and W‐IR. W‐NS and treatment groups demonstrated significantly lower stiffness than control groups. (B) Histological representation of CD31 immunohistochemical staining for all treatment conditions. (C) Green pixel count was used to quantify amount of CD31 immunofluorescence in each treatment group. No significant difference was found between W‐NS and W‐D100, though W‐DP was found to have a significantly lower pixel count than both groups (**p < 0.01 and p* < 0.05, respectively). Both W‐D100 (****p < 0.0001) and W‐DP (****p < 0.0001) were found to have higher CD31 immunofluorescence than W‐IR control. (D) Laser Doppler at wound closure in perfusion units was compared between all experimental groups. No significant difference was found between W‐NS and W‐D100 or between control groups W‐Vehicle and W‐IR. W‐D100 demonstrated higher perfusion units than W‐DP (*p < 0.05). W‐NS (****p < 0.0001), W‐D100 (****p < 0.0001), and W‐DP (**p < 0.01) had higher perfusion quantified by laser Doppler than W‐IR. (E) Representative images of Doppler scans of healed wounds are shown. (F) Laser Doppler perfusion as measurements in wound bed over duration of healing. DFO demonstrated elevated perfusion measures throughout course of healing more closely mimicking nonirradiated wounds relative to W‐Vehicle and W‐IR control groups.

In wounded skin, where mechanical testing was performed via biomechanical stress testing, DFO‐treated skin regardless of treatment type resulted in a non‐significant difference in stiffness compared to W‐NS. Significantly improved stiffness was found in W‐NS, W‐D100 and W‐DP compared to the irradiated control groups W‐IR and W‐Vehicle. No significant difference in stiffness was found between W‐IR and W‐Vehicle (Figure 6A).

### 3.7 DFO cream improves vascularity and perfusion in chronically irradiated excisional wounds

In wounded irradiated skin, quantification of CD31 staining demonstrated that W‐D100 had higher levels than W‐DP and both irradiated control groups. No significant difference in CD31 staining was found between W‐D100 and W‐NS. W‐DP also had significantly higher CD31 staining levels than W‐IR. Control groups W‐Vehicle and W‐IR had no significant difference in CD31 staining (Figure 6B,C).

Wounded skin at day seven, like irradiated unwounded skin, demonstrated a significant difference in perfusion units measured between W‐IR and the treatment groups W‐D100 and W‐DP (Figure 6D). These differences persisted at wound closure (Figure 6D–F). At wound closure, a significant difference was measured between W‐NS and W‐D100 compared to W‐Vehicle and W‐IR. The groups W‐NS and W‐D100 had significantly higher perfusion unit readings than W‐DP at wound closure, while groups W‐Vehicle and W‐IR had significantly lower perfusion unit readings at wound closure (Figure 6D–F). At all timepoints, no significant difference was found between W‐Vehicle and W‐IR; nor were there any differences between W‐NS and W‐D100.

## 4 DISCUSSION

The first aim of this study was to determine if the DFO cream formulation would adequately rescue RIF in irradiated, unwounded skin. Across all in vivo and ex vivo methodologies measured, both the D50 and D100 groups outperformed the IR control, including gross skin elasticity with the suction cutometer, cutaneous perfusion with the laser Doppler, and various histologic analyses. Lavin et al. previously demonstrated improvement in irradiated skin with either DFO patch delivery or DFO injection, which our present study parallels.^2^ Extending these findings, this study also shows that a cream formulation for DFO delivery does not appear to alter DFO effects on irradiated skin. The viscosity and molecular size of the individual compounds to create the cream modality were designed to allow for absorption of the cream itself as well as the DFO molecules into the cutaneous tissue. The results of this study confirm the ability to effectively deliver DFO in this new formulation to improve chronic radiation‐injured skin.

Determination of effect between D50 and D100 dosage was also undertaken, with comparison of these cream formulations to other previously studied and established DFO treatment methods for RIF of unwounded irradiated skin: a topical dermal patch (DP) and dermal injection (DI).^2^ When these treatment conditions were compared to one another in irradiated skin, D50 and DI had similar efficacy, with D50 modality slightly outperforming DI condition in a few metrics. D50 and DI were equivalent in in vivo elasticity, dermal thickness, collagen density and 8‐isoprostane assays. However, the D50 cohort had significantly improved perfusion compared to the DI cohort, as measured with both laser Doppler and CD31 staining.

Basket‐weave organization of collagen mirrors the native architecture of healthy skin and possesses robust mechanical properties. D50 was able to better maintain the basket‐weave organization of the extracellular matrix than DI, but less effectively than D100 or DP. D100 and DP were largely equivalent in their ability to improve cutaneous RIF. Between the D100 and DP groups, both conditions appeared similarly potent when analysing collagen density, dermal thickness, 8‐isprostane staining, and retention of basket‐weave collagen ultrastructure. D100 treatment showed some slight improvement in perfusion and CD31 expression compared to DP, perhaps indicating an improved angiogenic response with improved retention of the cream within the dermal and subcutaneous tissue compared the DP.

The second aim of this study sought to build upon these results by investigating the utility of the DFO cream formulation in irradiated wounded skin. Reflecting the results from irradiated unwounded skin, DFO treatment demonstrated improvements across all in vivo and ex vivo measurements, including cutaneous perfusion with the laser Doppler, various histologic analyses, scar stiffness via biomechanical testing, and speed of wound healing. The work of Lintel et al. comparing chronically irradiated wounds treated with DP formulation to untreated wounds used similar outcome measures, and these results were recapitulated by our findings.^13^ Interestingly, the D100 cream formulation was found to significantly outperform the DP formulation across several parameters, including speed of wound healing, Doppler perfusion at wound closure, and CD31 immunofluorescence. The two DFO formulations (D100 and DP) performed with no significant difference across measures including frequency of non‐healing wounds, wound thickness on H&E, collagen density on TC, scar tensile strength, and extracellular matrix analysis via Picrosirius Red staining.

Interestingly, our results demonstrate that DFO treatment of chronically irradiated skin results in a thinner dermis and less collagen deposition, while DFO treatment of chronically irradiated wounds results in a thicker dermis with more collagen deposition. These results recapitulate those from previous work that has studied topical DFO application in irradiated skin and chronically irradiated excisional wounds.^2^, ^4^, ^13^, ^28^ The reason for this difference in DFO's effect may relate to improvements in vascularity which could encourage remodelling of chronically scarred RIF‐affected skin. In the case of acute scarring in excisional wounds, a thicker dermis caused by increased collagen deposition may be evolutionarily preferable to enable expedient and effective wound closure. The perfusion encouraged by DFO treatment may contribute to the histological finding of irradiated wounds closing with architecture more similar to that of NS, an improvement that is particularly impactful in the thinner and more fragile wounds of irradiated skin which are susceptible to dehiscence.

Importantly, several limitations exist pertaining to our work. First, our experiment utilized only female mice. Although this allowed for control between experimental groups and data, there have been differences in the healing process identified between males and females. Future work should seek to validate our findings in male model organisms. Though care was taken to remove any superficial residue prior to performing laser Doppler and suction cutometer measurements, some remaining residue could still have been present during testing and affected the results. Residue could have remained from the cream itself or the adhesive bandages utilized to hold the treatments in place and keep the wounds clean. Furthermore, trauma to the skin and soft tissue from application of adhesives for the patch and cream treatment conditions or continuous injections for the DI group could alter the results of this experiment. With the experimental timeline concluding weeks after application of treatment, further research into longer‐term effects of these treatment modalities on RIF response would also be very informative. Additionally, investigation of the cream on normal, non‐irradiated skin was not investigated in this study and would serve as an important control to contextualize our findings. Finally, this experiment focused on the ability to rescue radiation‐induced, chronic fibrotic skin and improve wound healing in this setting. Application of these different DFO treatment modalities throughout different phases of radiation wounding and recovery would be an interesting area of application for further research.

## 5 CONCLUSION

The cream formulation of DFO demonstrates utility in both irradiated and excisionally wounded irradiated skin. This treatment can mitigate inflammation, promote reorganization of collagen in the extracellular matrix, and decrease dermal thickness to improve RIF in irradiated unwounded skin as effectively as the previously studied DFO patch formulation. However, in chronically irradiated skin, D100 DFO cream improves vascularity more than any other DFO formulation studied to date. Furthermore, in wounded skin, D100 treatment resulted in faster healing and improved perfusion compared to any other formulations. DFO cream could prove to be more user‐friendly in the clinical setting, especially in patients with severe complications following radiation, eliminating the need for any adhesive or bandage to assist with DFO application and greatly improving patient comfort and outcomes.

## AUTHOR CONTRIBUTIONS

**Charlotte E. Berry:** Conceptualization (lead); data curation (equal); formal analysis (equal); investigation (equal); methodology (equal); project administration (lead); resources (equal); software (equal); supervision (equal); validation (equal); visualization (equal); writing – original draft (lead); writing – review and editing (equal). **Darren B. Abbas:** Conceptualization (equal); data curation (equal); formal analysis (equal); funding acquisition (equal); investigation (equal); methodology (equal); project administration (equal); resources (equal); software (equal); supervision (equal); validation (equal); visualization (equal); writing – original draft (equal); writing – review and editing (equal). **Michelle Griffin:** Conceptualization (equal); formal analysis (equal); investigation (equal); methodology (equal); project administration (equal); supervision (equal); validation (equal); visualization (equal); writing – review and editing (equal). **Hendrik Lintel:** Investigation (equal); methodology (equal); writing – review and editing (equal). **Jason Guo:** Formal analysis (equal); supervision (equal); writing – review and editing (equal). **Lionel Kameni:** Investigation (supporting). **Andrew A. Churukian:** Investigation (supporting); methodology (supporting); project administration (supporting); writing – review and editing (supporting). **Alexander Z. Fazilat:** Investigation (supporting); methodology (supporting); project administration (supporting); writing – review and editing (supporting). **Kellen Chen:** Methodology (supporting); writing – original draft (supporting). **Geoffery C. Gurtner:** Conceptualization (supporting); funding acquisition (supporting); resources (supporting); supervision (supporting); writing – review and editing (supporting). **Michael T. Longaker:** Conceptualization (equal); funding acquisition (equal); resources (equal); supervision (equal); writing – review and editing (equal). **Arash Momeni:** Conceptualization (equal); supervision (equal); writing – review and editing (equal). **Derrick C. Wan:** Conceptualization (equal); data curation (equal); funding acquisition (equal); investigation (equal); resources (equal); supervision (equal); writing – original draft (equal); writing – review and editing (equal).

## FUNDING INFORMATION

This research was supported by the Center for Dental, Oral & Craniofacial Tissue & Organ Regeneration (C‐DOCTOR grant U24DE026914), NIH grants 1R01AR081343–01, 1R01DE032677–01, Wu Tsai Human Performance Alliance, and the Hagey Laboratory for Pediatric Regenerative Medicine.

## CONFLICT OF INTEREST STATEMENT

Michael T. Longaker and Geoffrey C. Gurtner have an equity stake in TauTona Group, the source from which the DFO Cream and Patches used in this experiment were purchased. Geoffrey C. Gurtner holds a patent for the treatment of chronic wounds with transdermal HIF‐1 modulators. Derrick C. Wan, Geoffrey C. Gurtner, and Michael T. Longaker hold a patent for the utilization of deferoxamine to condition irradiated tissue.

## Data Availability

The data underlying the results are available within the article and upon request to the corresponding author.
